# 

**Published:** 1790

**Authors:** 


### LONDON

### MEDICAL JOURNAL,

FOR THE YEAR 1790,

### PART THE FOURTH.

### CATALOGUE OF BOOKS.

1. AN Eſſay on the Scurvy: ſhewing effectual and practicable Means for its Prevention at Sea: with ſome Obſervations on Fevers; and Propoſals for the more effectual Preſervation of the Health of Seamen. By *Frederick Thomſon*, (a Surgeon in the Royal Navy) reſident at Kenſington. 8vo. *Robinſons*, London, 1790.

2. El Jornal Medico Londinenſe, traducido del Ingles al Caſtellano por Don *Thomas Hearn*, M. D. Medico revalidado por el real Tribunal de Proto-medicato de Madrid. 8vo. Cadiz, 1790.

3. Sammlung der neueſten Beobachtungen Engliſchen Aerzte und Wundarzte; aus dem Engliſchen. 8vo. Franckfort on the Main, 1789.

This is a German tranſlation of the London Medical Journal under a different title.

4. Prælectio Academica ſimplitiores et ſalubriores comprehendens de Febribus notiones; Auctore *Joſepho Pinilla Vizcayno*, Doct. Med. in Mayori Univerſali Gymnaſio Complutenſi, Regio Profeſſore primario, et Regalis Academiæ

Medicæ

Medicæ Matritensis Socio. 4to. Compluti, 1790.

5. *Horatii Bellini* Astensis Med. Doct. de Apoplexia tractatus medico-practicus; cui accedunt Commentaria textus 52, sect. 2, lib. 7, de judicationibus, et textus 17, sect. 2, cap. 3, prognosticorum Hippocratis. Tom. I. 8vo. Roma, 1790.

6. *Christiani Andreæ Cothenii*, Med. Doct. Dispositio Vegetabilium Methodica a Staminum numero desumta. 8vo. Berolini, 1790.

7. Delle lodi di Monsignor *Natale Saliceti*, Archiatro Pontificio; Orazione di *Pietro Pasqualoni*, recitata nell' Archiginnasio della Sapienza il di 2 Luglio, 1789. 4to. Roma, 1789.

8. La Chirurgia istantanea, in cui si tratta ancora della forenze; Volume due: di *Tommaso Maria Celoni*, Lettore di Notomia e Chirurgia, &c. 8vo. Roma, 1789.

9. Analisi Chimica delle acque dei bagni Pisani, e dell' acqua acidula di Asciano di *G. Santi*. 8vo. Pisa, 1789.

